# *Ab initio* Designed Antimicrobial Peptides Against Gram-Negative Bacteria

**DOI:** 10.3389/fmicb.2021.715246

**Published:** 2021-11-16

**Authors:** Shravani S. Bobde, Fahad M. Alsaab, Guangshuan Wang, Monique L. Van Hoek

**Affiliations:** ^1^School of Systems Biology, George Mason University, Manassas, VA, United States; ^2^College of Applied Medical Sciences, King Saud bin Abdulaziz University for Health Sciences, Al Ahsa, Saudi Arabia; ^3^Department of Pathology and Microbiology, University of Nebraska Medical Center, Omaha, NE, United States

**Keywords:** *ab initio*, antimicrobial peptide, design, computational prediction models, Gram-negative bacteria

## Abstract

Antimicrobial peptides (AMPs) are ubiquitous amongst living organisms and are part of the innate immune system with the ability to kill pathogens directly or indirectly by modulating the immune system. AMPs have potential as a novel therapeutic against bacteria due to their quick-acting mechanism of action that prevents bacteria from developing resistance. Additionally, there is a dire need for therapeutics with activity specifically against Gram-negative bacterial infections that are intrinsically difficult to treat, with or without acquired drug resistance. Development of new antibiotics has slowed in recent years and novel therapeutics (like AMPs) with a focus against Gram-negative bacteria are needed. We designed eight novel AMPs, termed PHNX peptides, using *ab initio* computational design (database filtering technology combined with the novel positional analysis on APD3 dataset of AMPs with activity against Gram-negative bacteria) and assessed their theoretical function using published machine learning algorithms, and finally, validated their activity in our laboratory. These AMPs were tested to establish their minimum inhibitory concentration (MIC) and half-maximal effective concentration (EC_50_) under CLSI methodology against antibiotic resistant and antibiotic susceptible *Escherichia coli* and *Staphylococcus aureus*. Laboratory-based experimental results were compared to computationally predicted activities for each of the peptides to ascertain the accuracy of the computational tools used. PHNX-1 demonstrated antibacterial activity (under high and low-salt conditions) against antibiotic resistant and susceptible strains of Gram-positive and Gram-negative bacteria and PHNX-4 to -8 demonstrated low-salt antibacterial activity only. The AMPs were then evaluated for cytotoxicity using hemolysis against human red blood cells and demonstrated some hemolysis which needs to be further evaluated. In this study, we successfully developed a design methodology to create synthetic AMPs with a narrow spectrum of activity where the PHNX AMPs demonstrated higher antibacterial activity against Gram-negative bacteria compared to Gram-positive bacteria. Thus, these peptides present novel synthetic peptides with a potential for therapeutic use. Based on our findings, we propose upfront selection of the peptide dataset for analysis, an additional step of positional analysis to add to the *ab initio* database filtering technology (DFT) method, and we present laboratory data on the novel, synthetically designed AMPs to validate the results of the computational approach. We aim to conduct future *in vivo* studies which could establish these AMPs for clinical use.

## Introduction

Antimicrobial peptides (AMPs) are evolutionarily conserved, small, cationic, amphiphilic molecules produced by prokaryotes and eukaryotes with antimicrobial and immunomodulatory properties (Lazzaro et al., 2020; Tornesello et al., 2020). AMPs are typically less than 50 amino acids in length, contain on average 41% hydrophobic residues and target the bacterial membrane enabling a fast mechanism of action preventing the organism from developing resistance (Browne et al., 2020). Overuse of antibiotics has led to a crisis due to the emergence of antibiotic-resistant bacteria. As we approach a post-antibiotic era, AMPs present therapeutic potential due to their proven broad-spectrum activity and characteristics that set them apart from traditional antibiotics (Browne et al., 2020; Cardoso et al., 2020).

Gram-negative bacteria have key structural differences from Gram-positive bacteria that make them intrinsically harder to eradicate. Gram-negative bacteria contain an outer membrane that protects the bacterium from environmental toxins and provides efflux out of the cell (Silhavy et al., 2010). This membrane permeability barrier has historically restricted the discovery of narrow-spectrum antibiotics against Gram-negative bacteria and this challenge is further compounded due to the rise in multi-drug resistance (MDR) strains resistant to multiple classes of antibiotics (Liu et al., 2019; Otsuka, 2020). Finally, Gram-negative bacteria can also use biofilms (a virulence factor) as a means to confer phenotypic antibiotic resistance which can make the bacteria up to 1,000-fold times more resistant when embedded in the exopolysaccharide matrix (Cepas et al., 2018). Thus, novel antimicrobials are needed to combat multi-drug resistant Gram-negative bacterial infections to decrease morbidity and mortality from these infections.

Computational approaches such as *in silico* machine-learning algorithm assisted motif identification using physiochemical properties (Okella et al., 2020), *de novo* design (Chen et al., 2019), rational *ab initio* design (Mishra and Wang, 2012), redesigning and optimizing existing AMPs (Torrent et al., 2011), quantitative structure-activity relationship (QSAR) computational modeling and screening (Cardoso et al., 2020) have been used to design AMPs; however, few synthetic AMPs have reached clinical therapeutic potential. Computational tools can be leveraged not only to design but also to predict AMP’s function as AMP discovery and testing *in vitro* can be time consuming as well as expensive. Prediction tools that use different AMP databases and multiple machine learning algorithms [support vector machines (SVMs), artificial neural networks (ANN), discriminate analysis (DA), random forest (RF), WEKA, and deep learning] can be used in conjunction with laboratory testing to identify AMPs with potential antimicrobial activity.

In this study, we used rational design (*ab initio* combined with the novel positional analysis) to develop narrow-spectrum synthetic AMPs against Gram-negative bacteria, predicted their function and properties using bioinformatics tools and tested the AMPs *in vitro* to assess their effects on the growth of antibiotic resistant and susception strains of *Escherichia coli* (*E. coli*) and *Staphylococcus aureus* (*S. aureus*) as well as potential toxicity against human red blood cells (RBCs) to assess their therapeutic potential. We hypothesized that the hypothesis-driven selection of input data for the computational design as well as improved ML-assisted antimicrobial peptide activity prediction will help us to develop and identify novel potent AMPs with higher activity against Gram-negative *E. coli* compared to *S. aureus*.

## Materials and Methods

### Antimicrobial Peptides Design

**FIGURE 1:**
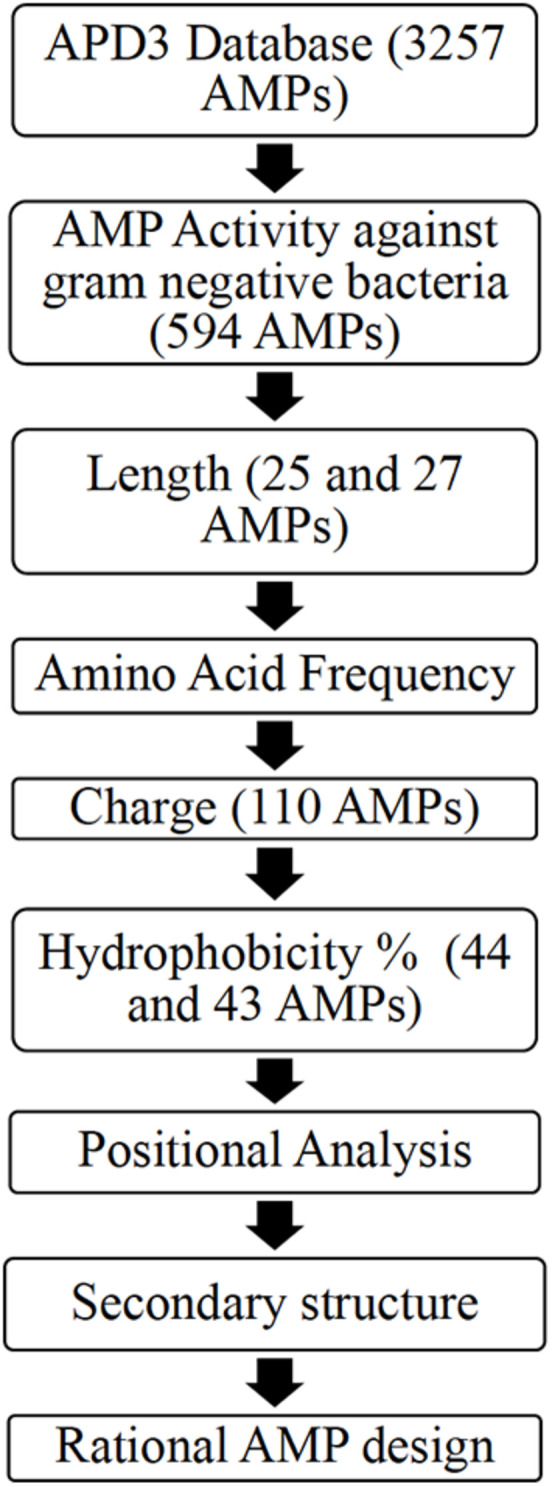
A flowchart describing the *ab initio* rational AMP design process for Dataset 1 including an additional filter: positional analysis [Adapted from Mishra and Wang (2012)].

*Ab initio* database filtering technology (DFT) developed by the laboratory of Dr. Guangshun Wang was used as the first step to design novel AMPs (Mishra and Wang, 2012). The database APD3 was used to obtain two AMP datasets (Wang et al., 2016). Dataset 1 (594 AMPs) included AMPs with reported activity against Gram-negative bacteria irrespective of their reported activity against Gram-positive bacteria. Dataset 2 (299 AMPs) included AMPs with reported activity against only Gram-negative bacteria (Wang et al., 2016). R-Studio (Package “Peptides”) and Microsoft Excel was used to obtain the properties of the AMPs dataset obtained from APD3 (Osorio et al., 2015; Microsoft Corporation, 2018; RStudio Team, 2020). Figure 1 illustrates the *ab initio* database filtering technology method used, where analyzing existing AMP properties is used to filter AMPs followed by rationally designing synthetic AMPs that meet the filtered criteria.

### PHNX-2 Through PHNX-5

**FIGURE 2:**
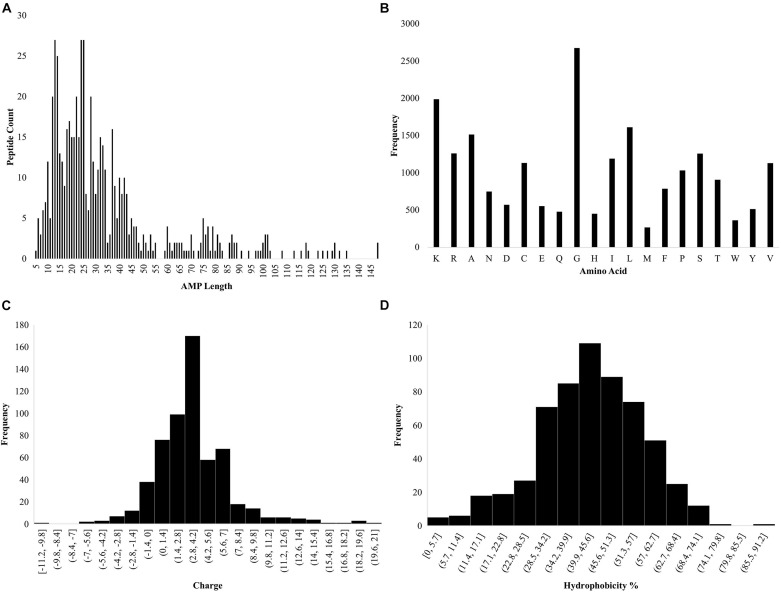
Statistics of AMP properties obtained from APD3. **(A)** Lengths of AMPs in Dataset 1. **(B)** Frequency of total amino acid residues in Dataset 1. **(C)** Charge of the AMPs in Dataset 1. **(D)** Hydrophobicity percentage of AMPs in Dataset 1 [hydrophobicity was calculated as a percentage of hydrophobic amino acids, based on the Kyte and Doolittle scale (Kyte and Doolittle, 1982) divided by the total number of amino acids per AMP].

*Ab initio* database filtering technology was used to design PHNX-2 through PHNX-5. The first filter used was length where the length of each peptide was calculated using the Excel formula “LEN” as shown in Figure 2A. From Dataset 1, the most commonly represented sequence length selected was 13 and 14 which occurred 27 and 25 times, respectively. Large AMPs > 20mer were initially not selected due to difficulty in their synthesis as well as the high likelihood of larger peptides forming complex 3-dimensional structures. The next filter we applied was the frequency of each amino acids in the entire dataset. MS Excel was used to conduct this analysis, the formula “LEN” was used where for example, =LEN(G2)-LEN(SUBSTITUTE(G2, “K,” “”) enabled Excel to calculate the number of times K occurs in each peptide. This was repeated for each amino acid in Dataset 1. Figure 2B illustrates the amino acid frequency in Dataset 1. Based on the *ab initio* method previously described by Mishra and Wang (2012) the amino acids were grouped as: non-polar hydrophobic (A, C, I, L, M, F, W, and V), polar uncharged (N, Q, S, T, and Y), small turn residues (G and P), acidic (D and E) and basic (K, R, and H) (Mishra and Wang, 2012). The next filter applied was charge and R-Studio “Peptides” package was used to obtain the charge of the AMPs (Osorio et al., 2015; RStudio Team, 2020). The code used was Charge (sequence, pH = 7, pKscale = “Lehninger”). The most commonly occurring charge in Dataset 1 was +4 (Figure 2C). The hydrophobicity of the amino acids was then measured using the *ab initio* method where the hydrophobicity percentage was calculated by dividing the total number of hydrophobic residues (based on the Kyte and Doolittle scale) by the total amino acids per AMP (Kyte and Doolittle, 1982; Mishra and Wang, 2012). In addition, tryptophan was also included by APD due to its strong interfacial preference. The most commonly occurring hydrophobicity percentages were 43 and 44% (Figure 2D). Thus, the major criteria for designing novel AMPs were obtained: peptide length of 13 or 14 amino acids with a +4 charge and 43–44% hydrophobicity. Thus, the designed AMP sequence should contain 5 and 6 hydrophobic residues for 13 and 14-residue long AMP, respectively. Furthermore at least 4 K (most frequently occurring positively charged amino acid in the dataset) will be needed to provide a +4 charge which would leave 4 residues in each sequence as the neutral G or S amino acids (both of which were equally represented in the dataset). Hence, a regular expression for a 13 residue AMP with 43–44% hydrophobicity, +4 charge to result in a helical secondary structure would be: F[IL][IL]K[IL][IL]KGGKGGK. The BLOSUM substitution matrix was used to substitute residues per position and the amino acid frequency per position was then used to rationally design PHNX-2 through PHNX-5 (Henikoff and Henikoff, 1992). At position 1, the amino acid F was chosen as it was the most frequent residue represented followed by I and R and resulted in designing synthetic AMPs with the highest score of predicted antimicrobial activity. The regular expression above was selected as the synthetic AMPs resulted in an alpha-helix structure to allow it to transverse the bacterial membrane.

### PHNX-1

After designing AMPs using the *ab initio* method, we wanted to use a purely data-based approach to develop a synthetic AMP; hence, we proposed combining DFT method with an additional filter termed positional analysis. This analysis consists of analyzing the residues at each position within a subset of AMPs to identify the most frequently occurring amino acid residue per position. Initially, a 20-residue length cutoff was established to prevent designing an AMP with structural complexities and 12-, 13-, and 14-residue length AMPs were selected from Dataset 1 as they occurred 20, 27, and 25 times, respectively. MS Excel was used to calculate the amino acid occurring most frequently per position to design a 14-residue long AMP called PHNX-1. To determine the amino acid per position, the Excel formula MID [$B2, COLUMNS ($B$2:C$2), position number] was used followed by COUNTIF ($D$2:$D$16, B19) to count the frequency of each residue per position. Finally, the formula MAX was used to calculate the residue that occurred most frequently per position. Table 1 illustrates the most frequently occurring amino acid per position by analyzing each position to design a 14-residue long AMPs in Dataset 1 which enabled the design of PHNX-1.

**TABLE 1:** Positional analysis results where the amino acid with the highest frequency per position was assessed and resulted in the design of PHNX-1.

**Position**	**Amino acid**	**Frequency**
1	Phenylalanine (F)	18
2	Leucine (L)	15
3	Leucine (L)	19
4	Lysine (K)	18
5	Isoleucine (I)	23
6	Valine (V)	14
7	Alanine (A)	10
8	Leucine (L)	11
9	Leucine (L)	21
10	Lysine (K)	14
11	Lysine (K)	21
12	Lysine (K)	20
13	Leucine (L)	16
14	Leucine (L)	10

### PHNX-6 Through PHNX-8

Dataset 2 was used to design PHNX-6, PHNX-7 and PHNX-8. Due to the excellent prediction results obtained for PHNX-1, positional analysis was again conducted, however, the 20-mer cut off used in the previous method was eliminated to ensure that an AMP with a potentially greater activity against Gram-negative bacteria was not excluded from this list. The most frequently occurring lengths in the entire dataset [13, 25, and 28 (frequency 13, 13, and 15, respectively)] were chosen from Dataset 2. Positional analysis (data shown in Supplementary Figure 1) was conducted on three separate subsets consisting of 13-, 25-, and 28-residue long AMPs to design PHNX-6, PHNX-7, and PHNX-8.

### Similarity and Properties of PHNX Antimicrobial Peptides

The similarity of the synthetic antimicrobial peptides to existing naturally or synthetically designed peptides was assessed using the APD3’s Calculation and Prediction tool and the AMPs with the greatest similarity were incorporated into Table 2 (Wang et al., 2016). APD3 tools^1^ were used to calculate the length, charge, Wimley–White whole-residue hydrophobicity of the peptide (the sum of whole-residue free energy of transfer of the peptide from water to POPC interface), Boman index, APD3 defined hydrophobicity ratio and Grand Average Hydropathy Value (GRAVY) of the designed AMPs where the FASTA format of the AMPs was input under “Calculation and Prediction,” ran and the properties were incorporated into Table 3 (Wang et al., 2016). The Hydrophobic moment (μH), Hydrophobicity (H) were obtained from HeliQuest^2^ where the AMP sequence was incorporated and the parameter window size was set as “Full” to obtain the two properties (Gautier et al., 2008).

**TABLE 2:** Synthetically designed sequences of the PHNX AMPs and their similarity to peptides within the APD3 database (Wang et al., 2016).

**Name**	**Sequence**	**Similarity**
PHNX-1	FLLKIVALLKKKLL	60% AP02977 (Temporin-PE)
PHNX-2	FGKLLKLGKGLGG	50% AP00739 (Caeridin-a1)
PHNX-3	FGKLLKLGKGLKG	50% AP03169 [Peptide LDKA (synthetic)]
PHNX-4	FLLKLGLGKKKLL	57.14% AP03112 [DFT503 (synthetic)]
PHNX-5	FLIKILKGGKGGK	50% AP02842 (Temporin-MS4)
PHNX-6	FIGAIASYLKKFR	69.23% AP00405 (Ranatuerin 6)
PHNX-7	GVVDIIKGAGKKFAKGLAGKI ANKK	62.96% AP02598 (Ocellatin-PT4)
PHNX-8	GLMDTVKNAAKNLAGQLLD KIKCKITG	96.42% AP01507 (Ranatuerin-2CPc)

**TABLE 3:** Calculated physicochemical properties of the synthetically designed peptides (PHNX-1 to -8).

Name	Length (n) (Wang et al., 2016)	Molecular Weight (Da) (Wang et al., 2016)	Charge (Wang et al., 2016)	Wimley–White whole-residue hydrophobicity (kcal/mol) (Wang et al., 2016)	Boman index (kcal/mol) (Wang et al., 2016)	GRAVY (Wang et al., 2016)	APD3 defined hydro-phobic ratio (%) (Wang et al., 2016)	Hydro-phobicity (H) (Gautier et al., 2008)	Hydro-phobic moment (μH) (Gautier et al., 2008)
**PHNX-1**	14	1640.20	4	−0.6	−1.5	1.46	71	0.81	0.41
**PHNX-2**	13	1287.60	3	−0.35	−0.82	0.33	38	0.43	0.53
**PHNX-3**	13	1358.72	4	0.63	−0.32	0.06	38	0.36	0.50
**PHNX-4**	13	1470.94	4	−0.51	−0.93	0.71	53	0.62	0.30
**PHNX-5**	13	1358.72	4	1.13	−0.32	0.17	38	0.37	0.34
**PHNX-6**	13	1513.84	3	−1.11	0.32	0.55	53	0.57	0.66
**PHNX-7**	25	2512.06	6	6.83	0.46	−0.02	44	0.17	0.35
**PHNX-8**	28	2947.57	3	6.15	0.8	0.04	46	0.34	0.37

### Bioinformatics Prediction of Antibacterial Activity

The following AMP prediction tools used to predict the antibacterial activity: AxPEP^3^ Deep-AmPEP30 and RF-AmPEP30 that uses two algorithms, convolutional neural network and random forest, respectively, to predict <30-residue AMPs; CAMP_*R3*_^4^ using the algorithms SVM, RF, Discriminate analysis and ANN; CLASSAMP^5^ algorithms SVM and RF and; DBAASP^6^ that uses a machine learning algorithm using the Moon and Fleming scale to assess the physiochemical properties of the AMPs (Hydrophobic moment, Charge density and depth-dependent potential) (Joseph et al., 2012; Waghu et al., 2016; Yan et al., 2020; Pirtskhalava et al., 2021). The FASTA formatted sequence of each AMP was input, run and the results were incorporated into Table 4.

**TABLE 4:** Bioinformatics prediction of antimicrobial activity potential of peptides PHNX 1-8.

Name	Predicted antimicrobial activity								
	AxPEP (Yan et al., 2020)		CAMP_*R3*_ (Waghu et al., 2016)				CLASSAMP (Joseph et al., 2012)		DBAASP (Pirtskhalava et al., 2021)
	Deep-AmPEP	RF-AmPEP	SVM	RF	ANN	DA	SVM	RF	
**PHNX-1**	0.92	0.97	0.99	0.98	AMP	1.00	0.98	0.99	AMP
**PHNX-2**	0.73	0.93	0.94	0.54	AMP	0.99	0.99	0.96	AMP
**PHNX-3**	0.77	0.95	0.98	0.73	AMP	1.00	0.99	0.97	AMP
**PHNX-4**	0.93	0.99	0.99	0.99	AMP	1.00	0.98	0.99	Non-AMP
**PHNX-5**	0.85	0.94	0.88	0.89	AMP	1.00	1.00	0.98	Non-AMP
**PHNX-6**	0.86	0.70	0.86	0.97	AMP	0.85	0.99	0.96	AMP
**PHNX-7**	0.92	0.84	1.00	1.00	AMP	1.00	Not	0.99	AMP
**PHNX-8**	0.89	0.88	0.93	1.00	AMP	0.98	Not	0.94	AMP

*Not, not antibacterial.*

### Bacterial Strains

*Staphylococcus aureus* ATCC 33592 (MDR) and BAA-1718, *E. coli* ATCC 51659 (MDR) and 4,157 were purchased from the American Type Culture Collection (Manassas, VA, United States). All strains are reference strains. Bacteria were grown in Tryptic Soy Broth (BD 211825), except *E. coli* 4157 which was grown in Nutrient Broth (Difco 234000), overnight in a shaking incubator (37°C). Bacteria were aliquoted, mixed with glycerol (final concentration of 20% of glycerol) and frozen at −80°C and enumerated via serial dilution and plating prior to experimentation.

### Peptide Synthesis

All peptides were synthesized to order by ChinaPeptides, Inc. (Shanghai, China) using Fmoc chemistry. Peptides were provided at >95% purity, and the purity and structure were confirmed with RP-HPLC and ESI-MS.

### Minimum Inhibitory Concentration Antimicrobial Activity Assay

The antibacterial screening test of the peptides (*n* = 3) was first determined at a final peptide concentration of 100 μg/ml and a final bacterial concentration of 5 × 10^5^ CFU/mL in Difco Mueller Hinton Broth (BD 275730) in a 96 well plate. The plate was incubated for 16–20 h at 37°C and then read on a spectrophotometer at OD600 nm. Peptides which showed full inhibition of growth at 100 μg/ml were taken forward for full MIC testing. Wells containing medium only were used as sterility control, in the wells around the edge of the plates. LL-37 was used as a control in the first run of experiments against the MDR bacterial strains (Turner et al., 1998). However, it showed >64 μg/ml against *S. aureus*. Hence, we searched for active alternative controls that are experimentally validated against MDR *E. coli* and *S. aureus* and selected IDR-1018 (Wieczorek et al., 2010; Jahnsen et al., 2013) and BF-CATH (Tajbakhsh et al., 2018). The sequence of BF-CATH was derived from DBAASP database listed as cathelicidin-BF-34 with the following sequence: KRFKKFFRKLKKSVKKRAKEFFKKPRVIGVSIPF (Tajbakhsh et al., 2018; Pirtskhalava et al., 2021). (*N.b.* This differs slightly from the sequence for BF-CATH in APD3. AP00896, KRFKKFF**K**KLKKSVKKRAKKFFKKPRVIGVSIPF).

The full range MIC activity testing of the peptides was then determined as previously reported using the Mueller Hinton Broth (BD 275730) in a 96 well plate following the CLSI protocol (Wiegand et al., 2008). Enumerated bacteria were diluted in MHB and 50 μl of 1 × 10^6^ CFU/mL (5 × 10^4^ CFU) was added to each well with 50 μl of a decreasing series of twofold peptide concentration. The plate was incubated for 16–20 h at 37°C and then read on a spectrophotometer at OD600 nm. Readings of less than 10% of the untreated control were marked as “clear wells” for calling the MIC (Schwarz et al., 2010). Student’s *t*-test was used to determine whether points were statistically different.

### EC_50_ Antimicrobial Activity Assay

The antimicrobial activity of the PHNX peptides (China Peptides custom synthesis) and LL-37 (AnaSpec 61302) against bacteria was determined as previously described (Barksdale et al., 2016, 2017). Briefly, 1 × 10^5^ CFU per well of bacteria were incubated with different peptide concentrations (*n* = 3) in a 50-μl solution of 10 mM sodium phosphate buffer (3 h, 37°C). Serial dilutions were then prepared in 1X Dulbecco’s PBS and 8 μl of each dilution was spotted in triplicate on Tryptic Soy Agar plates, which were incubated (37°C, 24 h) and CFUs were counted (Barksdale et al., 2016, 2017). Bacterial survival at each peptide concentration was calculated based on the ratio of the number of colonies on each experimental plate and the average number of colonies observed for assay cultures lacking peptide. The peptide concentration required to kill 50% of the viable bacteria in the assay cultures (EC_50_) was determined by plotting percent survival as a function of the log of peptide concentration (log μg/ml) and fitting the data using GraphPad Prism 6 (GraphPad Software Inc., San Diego, CA, United States). For the purpose of graphing, samples that had no peptide (0 μg/ml) are plotted at 1 × 10^–7^ μg/ml peptide. EC_50_ values were determined by fitting the data from the antimicrobial assays to a standard sigmoidal dose–response curve. Errors were reported as a standard error of the mean within 95% confidence interval of the deviation from the mean of the log EC_50_ values. Student’s *t*-test was used to determine whether points were statistically different. Wells containing only 10 mM sodium phosphate buffer were used as sterility control. IDR-1018 was used as a positive control in this experiment as it demonstrated activity under MIC conditions against the antibiotic-resistant strains.

### Hemolysis Assays

To measure the hemolytic activity of peptides, 2% human red blood cells were added to various dilutions of peptide reconstituted in PBS in a sterile U-bottom polystyrene 96 well plate (Dean et al., 2011). The commercially obtained, de-identified human RBCs (BIOIVT Westbury, NY) were prepared as follows: 1 mL of K2EDTA whole blood from a healthy donor was centrifuged at 1,600 *g* for 10 min and plasma was discarded. The remaining RBCs were washed 4 times with 1 ml 1× PBS (HyClone), then the pallet after the last wash was resuspended with 750 μl of 1× PBS. 2% RBCs suspension was prepared by adding 200 μl of washed cells to 9.8 mL of 1× PBS. 50 μl of 2% RBCs were added to each well (*n* = 3) containing diluted peptides resulting in final peptide concentration of 100, 10, and 1 μg/ml. 2% RBCs with 1× PBS alone served as the negative control (No peptide), and 2% RBC in deionized water as the positive control, leading to full lysis. The plate was incubated for 1 h at 37°C and then centrifuged at 1,000 rpm for 2 min. The supernatant was transferred to a fresh regular 96 well plate and read at OD540 as we have previously reported (Dean et al., 2011). Student’s *t*-test was used to determine whether points were statistically different.

## Results

### Similarity to Existing Antimicrobial Peptides

Due to the lack of new antimicrobials being developed to combat Gram-negative infections, we used computational approaches combined with traditional laboratory benchtop assays to develop and assess novel AMPs against drug resistant and antibiotic susceptible strains of Gram-negative bacteria. The *ab initio* database filtering technology (DFT) method (Mishra and Wang, 2012) combined with positional analysis was used on two datasets obtained from APD3, which resulted in the design of 8 novel, synthetic AMPs termed PHNX, referring to the Phoenix, a powerful bird arising from the ashes. The databases and methods used to design the peptides are described in the “Materials and Methods” section. Table 2 illustrates the sequences of the synthetically designed PHNX AMPs as well as their similarity to existing naturally occurring or synthetically designed AMPs in the APD3 database (Wang et al., 2016).

The similarity of PHNX AMPs to existing AMPs with proven antibacterial activity ranged between 50 and 96% for PHNX-1 through PHNX-8. The peptides PHNX-1, -2, and -5 through -8 were the most similar to naturally occurring AMPs, suggesting that they may be active against Gram-negative bacteria. PHNX-1 shares 60% similarity with Temporin-PE, a naturally occurring AMP found in the skin secretions of the common water frog (*Pelophylax* kl. *Esculentus*) with antimicrobial activity against *S. aureus* (MIC 2 μM), MRSA (MIC 4 μM) *E. coli* (MIC 16 μM), *Enterococcus faecalis* (*E. faecalis*) (MIC 8 μM), and *Candida albicans* (*C. albicans*) (MIC 4 μM)(Sang et al., 2018). Similarly, PHNX-2 and PHNX-5 shared 50% similarity with Caeridin-a1 (an AMP found in the skin secretions of the Australian White’s Tree Frog, *Litoria caerulea* with a diverse antimicrobial activity against *S. aureus* NCTC10788 (MIC 8 μM), MRSA NCTC12493 (MIC 16 μM), *E. faecalis* NCTC12697 (MIC 32 μM), *E. coli* NCTC10418 (MIC 32 μM), and *C. albicans* NCYC1467 (MIC 32 μM) and Temporin-MS4, an AMP found in a frog (*Hylarana maosuoensis*), with activity against Gram-positive bacteria *S. aureus* ATCC 25923 (MIC 9.4 μM), *E. faecalis* 981 (MIC 18.8 μM), and *Nocardia asteroides* 201118 (MIC 4.7 μM) (Wang X. et al., 2017; Li et al., 2018). PHNX-8 (designed using positional analysis on Dataset 2) had a high degree of similarity (96.42%) to a naturally occurring AMP Ranateurin 2CPc (Wang et al., 2016) found in the skin secretion of New World frog (*Lithobates capito*) (Conlon et al., 2009).

PHNX-6 and PHNX-7 were 69.23 and 62.96% similar to Ranateurin-6 and Ocellatin-PT4, respectively (Wang et al., 2016). Ranateurin-6 and Ocellatin-PT4 are AMPs found in the skin secretions of the American bull frog (*Rana catesbeiana*) and the ceara white-lipped frog (*Leptodactylus pustulatus*) with weak activity against Gram-positive *S. aureus* (MIC 100 μM) and Gram-negative *E. coli* ATCC 25922 (MIC 80 μM), with no hemolysis observed for either AMP (Goraya et al., 1998; Marani et al., 2015). PHNX-3 and PHNX-4 demonstrated similarity to a set of synthetically designed AMPs Peptide LDKA and DFT503. These AMPs were developed using Simulation-Guided Rational de Novo Design (Peptide LDKA) as well as *ab initio* design (DFT503), which is interestingly the same approach we used to design some of the PHNX AMPs (Chen et al., 2019; Mishra et al., 2019). Peptide LDKA has demonstrated activity against *E. coli* (MIC 35 μM) and *S. aureus* (10 μM) and interestingly also shares similarity to another *ab initio* designed AMP called DFTamP1 (Wang et al., 2016; Chen et al., 2019). DFT503 has demonstrated antibacterial activity against *S. aureus* USA300 LAC (MIC 3.1 μM), *S. aureus* M838-17, *Enterococcus faecium* V286-17 (anti-VRE, MIC 3.1–6.2 μM) with no activity against Gram-negative bacteria and also shares 71% similarity with DFTamP1 (Mishra et al., 2019).

All synthetically designed PHNX peptides expressed a minimum of 50% similarity to existing AMPs with proven antimicrobial activity further strengthening our argument that that there is a strong likelihood that PHNX AMPs will demonstrate activity *in vitro* but are still unique and novel peptides.

### PHNX Antimicrobial Peptides Properties

After assessing the similarity and differences of PHNX AMPs, the properties of the AMPs were calculated, and Table 3 summarizes the properties of the PHNX AMPs. Computational analyses have demonstrated that net charge and amphipathicity are the most important physiochemical properties that statistically differentiate anti-Gram-negative AMPs from others (Wang C. K. et al., 2017). AMPs designed using *ab initio* database filtering technology method (PHNX-2 to -5) were each 13 residues long and AMPs designed using positional analysis (PHNX-1, PHNX-6, PHNX-7, and PHNX-8) were 14, 13, 25, and 28 residues long, respectively. All PHNX AMPs were positively charged (to allow them to associate with the negatively charged bacterial membrane) with the majority of the peptides having a charge of +3 and +4 and an outlier of +6 observed in PHNX-7. Studies have demonstrated that high cationicity in synthetically designed AMPs correlate with increased *in vitro* antibacterial activity and minimal cytotoxicity up to a threshold of +8 beyond which an increase in hemolytic activity is observed (Bahar and Ren, 2013; Lee et al., 2019). However, lower cationicity has been associated with *in vivo* active peptides (Mishra et al., 2019). Thus, the PHNX AMPs with the cationic charges between +3 through +6 will hopefully exhibit antimicrobial effects with minimal hemolytic activity.

The ideal AMP needs to be amphipathic to allow transport in an aqueous environment and interaction with bacterial lipid bilayer membrane. Hence, we used six separate measures to assess the PHNX AMPs hydrophobicity and amphipathicity including: the Wimley–White hydrophobicity, Hydrophobic moment <μH>, Hydrophobicity, Grand average of hydropathicity index score, the Boman index to assess protein binding potential and finally, the APD3 defined hydrophobic ratio. The Wimley–White whole-residue hydrophobicity was positive for PHNX-5, PHNX-7, and PHNX-8 and negative for others indicating that PHNX AMPs ranged between neutral to slightly hydrophilic (Wimley and White, 1996). Additionally, PHNX AMPs (except PHNX-7) had positive GRAVY scores indicating hydrophobicity; however, the scores were relatively close to neutral suggesting some amphipathicity. PHNX-1 had the highest GRAVY score of 1.46.

The Boman index, which assesses protein binding potential, was negative for PHNX-1 to -5 indicating hydrophobicity but positive for PHNX-6 to -8. It should be noted, however, that all Boman index scores (positive or negative) were near 0 for all AMPs except PHNX-1 (−1.5 kcal/mol). This was expected as AMPs typically do not bind other proteins but instead transverse and disrupt the bilayer membrane leading to membrane rupture. The APD-defined “hydrophobic ratio” was 38% for rationally PHNX-2, -3, and -5 which were all *ab initio* designed AMPs. PHNX-4, also an *ab initio* designed AMP, had a higher hydrophobic ratio of 53%. PHNX-1 had the highest hydrophobic ratio of 71% and the ratio for the longer AMPs, PHNX-7 and -8, was 44 and 46%, respectively. PHNX-6, designed using positional analysis, had a hydrophobic ratio of 46%. The APD-defined hydrophobic ratio ordered the peptides similarly to the calculation of H, the “Hydrophobicity” calculation for the peptides whose values range from −1.01 to 2.25 (Gautier et al., 2008). PHNX-1 and -4 had the highest scores (0.81 and 0.62, respectively) and however, all the AMPs had scores <1 indicating amphipathicity. The hydrophobic moment, μH, a value between 0 and 3.26 which indicates the amphipathicity of the AMPs perpendicular to its axis (Gautier et al., 2008) was higher than 0.5 for PHNX-2, -3, and -6. The other PHNX AMPs had a lower hydrophobic moment where PHNX-1 was 0.41 μH and PHNX-4, -7, and -8 had a hydrophobic moment below 0.4 μH.

Overall, we observed a diversity in APD3-defined hydrophobic ratios with PHNX-4 and PHNX-1 as the two outliers, with high ratios compared to the other AMPs designed rationally or using positional analysis. Based on the other five statistics to measure the hydrophobicity, PHNX AMPs overall are likely amphipathic structures with low protein binding potential (evidenced by the Boman index), neutral to slightly hydrophilic AMPs (Wimley–White whole-residue hydrophobicity), hydrophobic but close to neutral GRAVY and a Hydrophobicity (H) score ranging close to neutral indicating amphipathicity. The amphipathicity of the peptides can also be observed in the measurements of the hydrophobic moment (μH, Table 3) and in the helical wheel projections shown in Supplementary Table 1.

### PHNX Antimicrobial Peptides Antimicrobial Activity Prediction

A variety of prediction tools including different machine learning algorithms [support vector machine (SVM), random forest (RF), artificial neural network (ANN) and discriminate analysis (DA)] were used to assess PHNX AMPs predicted antimicrobial activity. Using a diverse set of tools ensured that the PHNX AMPs function was assessed against a variety of AMP datasets to increase the power of the prediction. The antimicrobial activity was predicted using four prediction tools across nine algorithms with the resulting scores listed in Table 4. PHNX-1 scored high (probability ≥ 0.92) for antimicrobial activity across all prediction tools and algorithms. PHNX-2, -3, and -6 were predicted as AMPs but had at least one score below 0.85 (PHNX-2 scored 0.73 and 0.54 using Deep-AmPEP and CAMP_*R3*_-RF; PHNX-3 scored 0.77 and 0.73 using Deep-AmPEP and CAMP_*R3*_-RF and PHNX-6 scored 0.70 using RF-AmPEP). The other PHNX AMPs were predicted as non-AMPs or not antimicrobial by at least one out of the nine predicting algorithms. PHNX-4 and -5 were predicted as non-AMP by DBAASP which uses a machine learning algorithm based on physio-chemical properties of AMPs but scored >0.85 as predicted by the other algorithms (Pirtskhalava et al., 2021). PHNX-7 and -8 were classified as not antimicrobial by CLASSAMP, however, PHNX-8 was predicted to be an AMP with a probability > 0.85 using other algorithms and PHNX-7 scored ≥ 0.84 by all algorithms.

Overall, 100% of PHNX AMPs were predicted to be AMPs by at least seven of the nine algorithms, 75% predicted as AMPs by eight algorithms and 12.5% predicted by all 9 algorithms. Thus, we can conclude it is likely that all PHNX AMPs, especially PHNX-1, are antimicrobial, with antibacterial activity, based on the scores established by multiple algorithms that use different training datasets to make their predictions.

### PHNX *in vitro* Inhibition of Representative Gram-Positive and Gram-Negative Bacteria

We then tested the *in vitro* inhibition of MDR and antibiotic-susceptible *E. coli* and *S. aureus* strains against the PHNX AMPs. These two bacteria are frequently utilized as model organisms to test the activity of AMPs and were selected to demonstrate whether the designed peptides met the design criteria of anti-Gram-negative activity, as well as the broader “antibacterial” activity.

#### Screening

**FIGURE 3:**
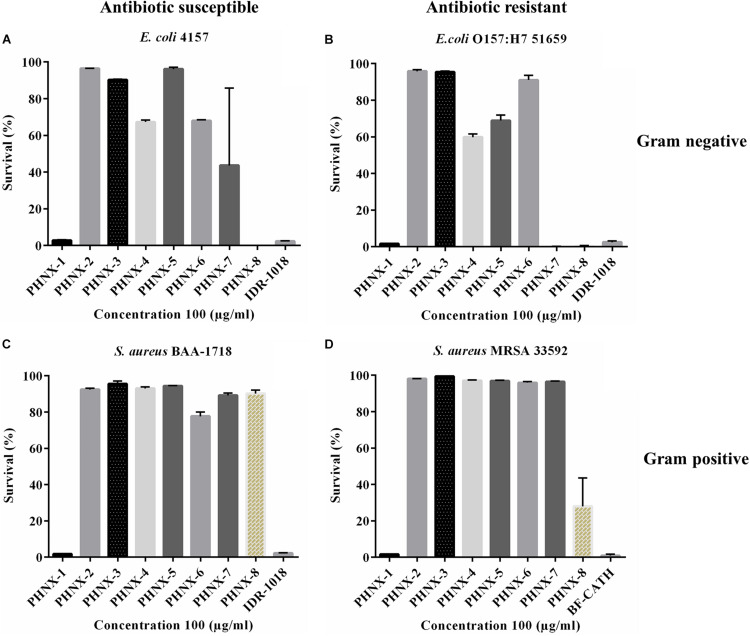
Antibacterial screening of PHNX-peptides and controls (IDR-1018 and BF-CATH) at 100 μg/ml against. **(A)** Antibiotic susceptible *Escherichia coli* 4157. **(B)** *Staphylococcus aureus* BAA-1718. **(C)** Antibiotic resistant *E. coli* O157:H7 51659. **(D)** *S. aureus* MRSA 33592 where PHNX-1 led to almost 0% bacterial survival against all strains and PHNX-7 and -8 against the Gram-negative bacteria.

The peptides were first screened at a high concentration of 100 μg/ml against MDR and antibiotic susceptible strains, and the active peptide against these bacteria qualified for minimal inhibitory concentration (MIC) determination (Figure 3). IDR-1018 and BF-CATH were used as controls in screening against MDR *E. coli* and *S. aureus* where the two control AMPs demonstrated 100% bactericidal activity at 100 μg/ml concentration as shown in Figure 3. We then assessed the MIC of the following PHNX AMPs, PHNX-1 which inhibited the growth of all bacterial strains, PHNX-8 which showed activity against the *E. coli* strains. PHNX-7 slightly inhibited MDR *E. coli* 51659, however, all other AMPs demonstrated no antibacterial activity against the MDR or antibiotic susceptible strains in the screen.

#### Minimum Inhibitory Concentration

**FIGURE 4:**
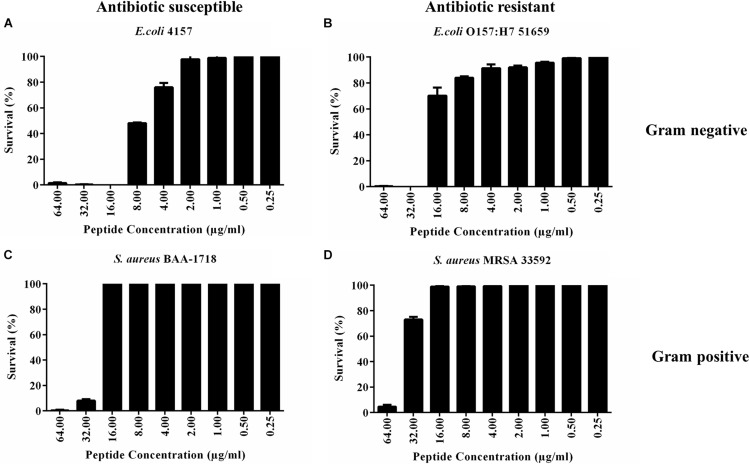
Minimum inhibitory concentration (MIC) of PHNX-1 against four strains of bacteria. **(A)** Antibiotic susceptible *E. coli* 4157 (MIC = 16 μg/ml). **(B)** *E. coli* O157:H7 51659 (MIC = 32 μg/ml). **(C)** *S. aureus* BAA-1718 (MIC = 32 μg/ml). **(D)** *S. aureus* 33592 (MIC = 64 μg/ml).

PHNX-1, with the predicted score of ≥0.92 probability of having antimicrobial activity, had a MIC of 32 and 64 μg/ml against MDR *E. coli* 51659 and *S. aureus* 33592, respectively, as demonstrated in Figure 4. Antibiotic susceptible strains were sensitive to PHNX-1 with a MIC of 16 μg/ml against *E. coli* 4157 and 32 μg/ml against *S. aureus* BAA-1718. PHNX-7 exhibited a high MIC value of >64 μg/ml against MDR *E. coli* 51659. PHNX-8 had a MIC of 64 μg/ml against MDR *E. coli* 51659 and a MIC of 32 μg/ml against antibiotic susceptible strain of *E. coli* 4157 without any activity against the antibiotic resistant or susceptible strains of Gram-positive bacterium *S. aureus*. Table 5 presents the MIC values of all the PHNX peptides against drug resistant and antibiotic susceptible strains of *E. coli* and *S. aureus* where LL-37, BF-CATH and IDR-1018 were used as the control AMPs against the four bacterial strains, as they have known activity against these bacteria. Although we used different strains, BF-CATH showed a strong antimicrobial activity against *E. coli*, similar to the levels previously reported of 2.3 μg/ml against *E. coli* ATCC 25922 and it was not very effective against *S. aureus* (Wang et al., 2008). Similarly, IDR-1018 showed good activity against the strains tested here (∼16 μg/ml), similar to previously reported activities (Wieczorek et al., 2010; Jahnsen et al., 2013). Additionally, while LL-37 was initially selected for use as a control against antibiotic-resistant strains of *E. coli* and *S. aureus*, our data demonstrated poor antimicrobial activity of >64 μg/ml against MRSA, similar to previously reported activities (Turner et al., 1998). We added a column to Table 5 summarizing the AMP predictions from Table 4 to enable comparisons between the predictions and the *in vitro* experimental data.

**TABLE 5:** Minimum inhibitory concentration (MIC) of PHNX peptides (μg/ml) against multi-drug resistant and antibiotic susceptible strains of *E. coli* and *S. aureus*.

Bacteria peptides	*E. coli* ATCC 51659	*S. aureus* ATCC 33592	*E. coli* ATCC 4157	*S. aureus* ATCC BAA-1718	Consensus from predictors (Table 4)
**PHNX-1**	32	64	16	32	Active (probability ≥ 0.92)
**PHNX-2**	>100	>100	>100	>100	Mixed predictions (see text, 0.54 by CAMP_*R3*_-RF)
**PHNX-3**	>100	>100	>100	>100	Mixed predictions (see text, 0.73 by CAMP_*R3*_-RF)
**PHNX-4**	>100	>100	>100	>100	Active (except by DBAASP)
**PHNX-5**	>100	>100	>100	>100	Active (except by DBAASP)
**PHNX-6**	>100	>100	>100	>100	Mixed predictions (0.70 RF-AmPEP)
**PHNX-7**	>64	>100	>100	>100	Active (except by CLASSAMP-SVM)
**PHNX-8**	64	>100	32	>100	Active (except by CLASSAMP-SVM)
**LL-37**	32	>64	NT	NT	
**BF-CATH**	8	64	4	NT	
**IDR-1018**	16	16	NT	16	

*NT refers to not tested. LL-37, BF-CATH, and IDR-1018 were control peptides tested against the bacterial strains. The consensus from the antimicrobial peptide predictors in Table 4 is indicated in the last column for comparison.*

#### EC_50_ Results

**FIGURE 5:**
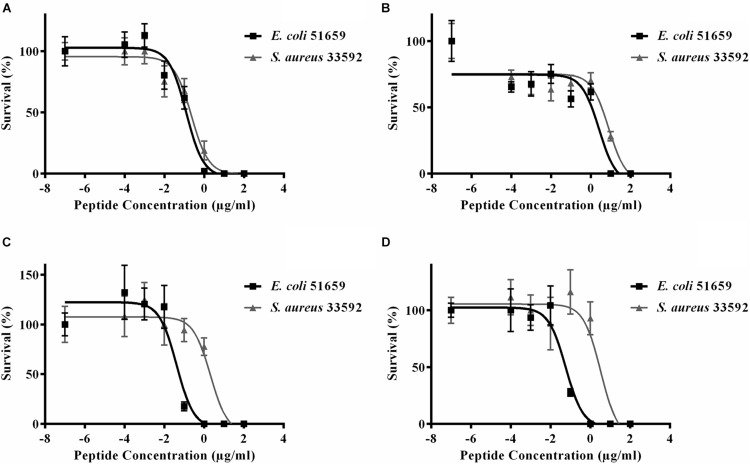
Half maximal effective concentration (EC_50_) of **(A)** PHNX-1, **(B)** PHNX-6, **(C)** PHNX-7, and **(D)** PHNX-8 against antibiotic resistant *E. coli* O157:H7 51659 and *S. aureus* MRSA 33592.

We next tested the PHNX AMPs against drug-resistant strains of *E. coli* and *S. aureus* under low-salt conditions to eliminate the probability of salt-mediated inactivation which would prevent the AMP from inhibiting bacterial growth. Interestingly, all PHNX AMPs, except PHNX-2 and -3, demonstrated activity inhibiting the growth of MDR *E. coli* and MRSA under low-salt conditions. These assays determine the half-maximal effective concentration (EC_50_) for antibacterial activity under 10 mM sodium phosphate conditions, following published protocols (Barksdale et al., 2016). PHNX-1 demonstrated the highest activity, as expected based on the MIC and bioinformatic prediction results, with an EC_50_ of 0.12 and 9.22 μg/ml against MDR *E. coli* and MRSA, respectively (Figure 5). PHNX-7, a highly cationic AMP, and PHNX-8, the longest AMP with a C-terminal rana box, demonstrated an EC_50_ of 0.04 and 0.06 μg/ml against MDR *E. coli* and 2.09 and 3.31 μg/ml against MRSA, respectively. PHNX-6, also designed using positional analysis, demonstrated an EC_50_ of 2.60 and 7.95 μg/ml against MDR *E. coli* and MRSA. Finally, PHNX-4 and PHNX-5 demonstrated an EC_50_ of 2.91 and 4.95 μg/ml against MDR *E. coli* and 4.85 and >10 μg/ml against MRSA. It should also be noted that PHNX-7 and -8, designed using positional analysis on Dataset 2, demonstrated a lower EC_50_ against Gram-negative MDR *E. coli* compared to the Gram-positive *S. aureus*, as shown in Figure 5, further confirming our hypothesis that using a “Gram-negative only” dataset resulted in rationally designing AMPs with higher activity against Gram-negative bacteria. Table 6 outlines the EC_50_ results of the MDR *E. coli* and *S. aureus* strains against all the 8 PHNX peptides including the 95% confidence intervals, and the EC_50_ results also presented in micromolar (μM) units. PHNX-4, -5, -6, and -8 had a larger range within the 95% confidence interval of EC_50_ against MRSA (for PHNX-4, -6, and -8) and *E. coli* (for PHNX-5) indicating greater variability in the concentration of the peptide required to inhibit bacterial growth and thus, increased likelihood of variable activity for the above peptides.

**TABLE 6:** Half maximal effective concentration (EC_50_) of PHNX peptides against multi-drug resistant strains of *E. coli* and *S. aureus*.

Peptides	Bacteria	EC_50_ (μg/ml)	95% CI (μg/ml)	EC_50_ (μM)	Consensus from predictors (Table 4)
**PHNX-1**	*E. coli* ATCC 51659	0.12	0.06–0.3	0.08	Active (probability ≥ 0.92)
	*S. aureus* ATCC 33592	0.22	0.10–0.52	0.14	
**PHNX-2**	*E. coli* ATCC 51659	>10	N/A	>7.78	Mixed predictions (see text, 0.54 by CAMP_*R3*_-RF)
	*S. aureus* ATCC 33592	>10	N/A	>7.78	
**PHNX-3**	*E. coli* ATCC 51659	>10	N/A	>7.34	Mixed predictions (see text, 0.73 by CAMP_*R3*_-RF)
	*S. aureus* ATCC 33592	>10	N/A	>7.34	
**PHNX-4**	*E. coli* ATCC 51659	2.91	1.30–6.53	1.98	Active (except by DBAASP)
	*S. aureus* ATCC 33592	4.85	1.90–12.36	3.29	
**PHNX-5**	*E. coli* ATCC 51659	4.95	1.90–12.91	3.64	Active (except by DBAASP)
	*S. aureus* ATCC 33592	>10	N/A	>7.36	
**PHNX-6**	*E. coli* ATCC 51659	2.60	0.90–7.57	1.72	Mixed predictions (0.70 RF-AmPEP)
	*S. aureus* ATCC 33592	7.94	2.85–22.13	5.24	
**PHNX-7**	*E. coli* ATCC 51659	0.04	0.02–0.12	0.02	Active (except by CLASSAMP-SVM)
	*S. aureus* ATCC 33592	2.09	0.61–7.10	0.83	
**PHNX-8**	*E. coli* ATCC 51659	0.06	0.02–0.14	0.02	Active (except by CLASSAMP-SVM)
	*S. aureus* ATCC 33592	3.31	0.89–12.29	1.12	

*N/A refers to not applicable where the 95% Confidence Interval could not be calculated. IDR-1018 was used as a control against *E. coli* and *S. aureus* and demonstrated bacterial killing at 100 μg/ml.*

### Hemolytic Activity and Therapeutic Potential of the PHNX Antimicrobial Peptides

**FIGURE 6:**
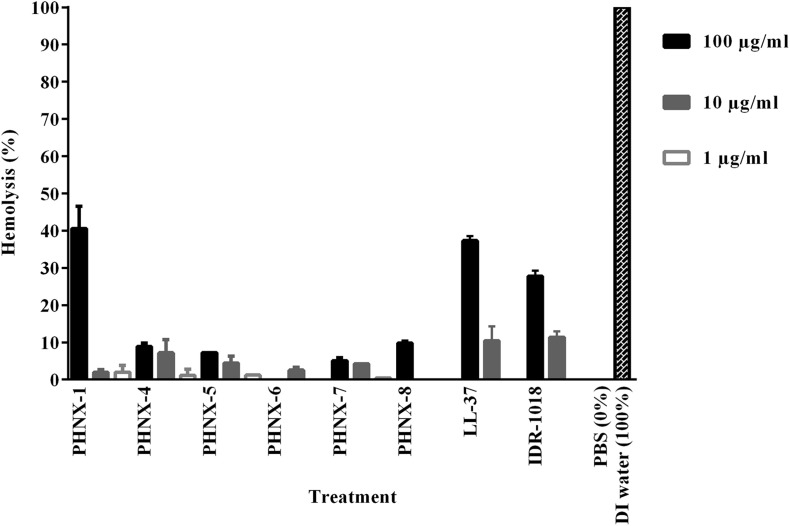
Hemolysis of the PHNX peptides against human red blood cells collected in EDTA with LL-37, deionized (DI) water and IDR-1018 were used as the controls. All PHX AMPs demonstrated hemolytic activity not significantly different from or lower than the controls.

After establishing MIC and EC_50_ activity, we tested the hemolytic potential of the synthetically designed PHNX AMPs. PHNX-1 demonstrated the highest hemolytic activity (approximative 40% hemolysis) at 100 μg/ml with minimal hemolysis at 10 and 1 μg/ml. PHNX-4, -5, -7, and -8 demonstrated higher hemolytic activity at 100 μg/ml sequentially decreasing at 10 and 1 μg/ml. LL-37 and IDR-1018 were the two controls used against human red blood cells (RBCs) to test hemolysis and demonstrated a similar pattern sequentially reducing hemolytic activity with approximately 40% hemolysis at 100 μg/ml, 10% at 10 μg/ml and negligible hemolysis at 1 μg/ml. This level of hemolysis is significantly higher than is seen with defribrinated sheep and horse blood, which may reflect the fragility of the RBCs collected in EDTA as an anticoagulant. Since the PHNX peptides showed antimicrobial activity between 16 and 64 μg/ml, this range is covered by the hemolysis testing. All PHNX AMPs outperformed the control peptides with overall lower hemolytic activity at each of the three concentrations. Figure 6 demonstrates the hemolytic activity of PHNX AMPs. As the hemolytic concentrations are not significantly different than the MIC concentrations (within an order of magnitude), these PHNX peptides probably need additional development in future work to increase their therapeutic index. Additionally, future work on using the MIC concentration of each AMP with a narrower range of AMP concentrations to assess cytotoxicity would result promising results. The therapeutic index may also serve as an *in vitro* filter to identify the peptides with the best cell selectivity. A high therapeutic index indicates high safety and minimal toxicity *in vivo* (Jiang et al., 2014; Mahlapuu et al., 2016; Wang C. K. et al., 2017).

## Discussion

In this new method, *ab initio* database filtering technology (DFT) was combined with a new step of positional analysis to computationally design novel synthetic antimicrobial peptides (AMPs) termed PHNX AMPs. The APD3 dataset of AMPs was parsed to generate a set of peptides with a narrow spectrum of activity against Gram-negative bacteria. This dataset was used in our computational approach to design new peptides with the hypothesis that the resulting peptides would have activity against Gram-negative bacteria. Our results show that 3/8 peptides (PHNX-1, -7, and –8) have antimicrobial activity against Gram-negative bacteria at 100 μg/ml or less and 6/8 (PHNX-1, -4, -5, -6, -7, and -8) have activity against Gram-negative bacteria under low-salt conditions (EC_50_) at 10 μg/ml or less. These results support our hypothesis that designing peptides by this method to be active against Gram-negative bacteria leads to a high number of peptides with that desired activity.

Overall, the PHNX AMPs demonstrated >50% similarity to existing AMPs (naturally occurring or synthetically designed) with computed properties traditionally observed in AMPs with antibacterial activity. PHNX-7 and PHNX-8 were the two outliers, PHNX-7 had high cationicity which predicted higher antibacterial activity with low hemolytic potential and PHNX-8, an AMP uniquely characterized by a “Rana box” which consists of a cyclic disulfide bridge at the C-terminus due to flanking cysteine residues separated by other four or five residues (Bao et al., 2018). AMPs with the Rana box domain share a structural analogy with Polymyxin, a cyclic peptide antibiotic used to treat multi-drug resistant infections caused by *Pseudomonas* spp. and *Acinetobacter* spp. where studies have demonstrated the importance of the structure within the Rana box which may correlate with an AMPs antimicrobial activity (Kozić et al., 2015). This correlated with our results where PHNX-7 and -8 had the lowest EC_50_ and the highest activity against Gram-negative organisms compared to all other AMPs.

We further hypothesized that measures of hydrophobicity and amphipathicity are important for activity. PHNX-1 (0.41 μH) had a hydrophobic moment closer to the Apo AMPs we previously discovered, which have also demonstrated antibacterial activity (Barksdale et al., 2016). In the data we obtained however, no obvious correlation was observed between the hydrophobicity and amphipathicity scores and the PHNX AMP’s *in vitro* activity. The “APD3 defined hydrophobic ratio (%)” somewhat correlated with EC_50_ activity, as most of the peptides with EC_50_ activity had greater than 40% APD3 defined hydrophobic ratio (%). It is generally thought that amphipathic AMPs typically demonstrate antibacterial activity, especially if helical. Some AMPs, like LL-37, can be membrane-active where the AMPs are disordered until interacting with the bacterial membrane to form an amphipathic helix penetrating the bacterial membranes (Hollmann et al., 2018). The mechanism of action for each PHNX AMP has not yet been determined.

Bioinformatics based AMP prediction can be complex as each prediction tool uses a different algorithm with a different training dataset which can result in a diversity of predicted AMP probabilities. Hence, we used nine different predictors and although all PHNX AMPs were predicted as antimicrobial, *in vitro* testing demonstrated that only PHNX-1 and PHNX-8 had antibacterial activity under high-salt conditions against the MDR strain of *E. coli*. However, it should be noted that PHNX-1 demonstrated the highest probability of having antimicrobial activity across all predictors with the highest overall consensus for predicted activity. This reflected our laboratory results where PHNX-1 demonstrated antibacterial activity under both MIC and EC_50_ conditions against Gram-positive and Gram-negative bacteria. Under low-salt conditions, all AMPs, except PHNX-2 and -3, demonstrated activity against the drug resistant strains of *E. coli* and *S. aureus* and no PHNX AMP was hemolytic at 10 μg/ml. PHNX-2 and -3 had the lowest probability of antimicrobial activity as predicted by Deep-AmPEP and CAMP_*R3*_-RF algorithms compared to the other PHNX AMPs, and the lab results demonstrated no activity against any bacterial strains. Prior studies have established CAMP_*R3*_-RF as the analytical tool with the best performance due to a large training dataset resulting in high accuracy in its predictions and our study further corroborated that result (Gabere and Noble, 2017). The EC_50_ results also imply that salt-mediated inactivation may have played a role in preventing the PHNX AMPs from inhibiting bacterial growth under high salt conditions. Studies have demonstrated that serum and salt mediated inactivation has prevented antibacterial activity of peptides *in vivo* with short amphipathic helical AMPs having the highest likelihood of salt-resistance (Mohanram and Bhattacharjya, 2016). PHNX-1, a short helical AMP, demonstrated activity under high salt conditions and thus presents as a candidate with expected *in vivo* activity and the potential for further development as a potential clinical therapeutic.

Overall, the PHNX AMPs designed using the novel positional analysis method (PHNX-1, -6, -7, and -8) demonstrated higher antibacterial activity against Gram-negative bacteria than the AMPs designed using only the *ab initio* DFT method (PHNX-2 to -5). In our study, using the DFT approach, the BLOSUM substitution matrix was used to substitute amino acids with equal frequency within the dataset; however, these substitutions may not have resulted in equivalent activity. Additionally, our approach in designing these AMPs combined a rational design element with positional analysis; for example, the decision to choose F at position 1. Additional residue substitution at this position may improve *in vitro* activity. PHNX-1 and PHNX-6 through -8 were designed using positional analysis method (frequency per position), which is a data-only approach and resulted in demonstrated *in vitro* activity against the MDR bacterial strains. Our hypothesis that using a dataset of AMPs with activity against Gram-negative bacteria would enable the *ab initio* design of synthetic peptides with higher activity against Gram-negative bacteria was proven. All PHNX AMPs demonstrated slightly higher activity against MDR and antibiotic susceptible strains of Gram-negative *E. coli* compared to the Gram-positive *S. aureus*. Although PHNX AMPs were active against Gram-positive *S. aureus*, every AMP had better activity against *E. coli*, supporting our design approach of designing a Gram-negative active peptide. PHNX-1 had a lower MIC against *E. coli* when compared to *S. aureus* and PHNX-8 had no activity against *S. aureus* but demonstrated reasonable MIC results against the Gram-negative *E. coli*. The MIC results were further supported under low-salt EC_50_ conditions where PHNX-1 and PHNX-4 though -8 demonstrated uniformly lower EC_50_ results against the Gram-negative MDR strain when compared to the Gram-positive MDR strain.

We have thus established a method to computationally design AMPs against a narrow spectrum class of bacteria (Gram-negative). In future work, these results against MDR and antibiotic susceptible bacteria need to be broadened and further corroborated by testing against other Gram-negative bacteria such as *Pseudomonas* and *Klebsiella*. This study confirms that the database filtering technology is likely to generate peptides with desired activity by starting from a defined set of candidates annotated in the APD3 (Mishra and Wang, 2012; Wang et al., 2016). Different from the original study where the most probable parameters were used to design a potent peptide against mainly Gram-positive methicillin-resistant *S. aureus* (MRSA), our designed peptides are primarily active against Gram-negative bacteria based on the inclusion criteria in the original datasets. Of note is that the peptides designed against Gram-positive bacteria have less cationic amino acids than the peptides we designed here. In the original DFT approach, the anti-Gram-positive peptides were generally of +1 charge, while, for our approach, the peptides most commonly had a charge of +4. This is further supported by recent work by Mishra et al. (2019), who found that peptides with higher net positive charge but with low number hydrophobic amino acids were more likely to be active against Gram-negative bacteria *in vivo*. Highly cationic synthetically designed AMPs (up to +8) have been found to correlate with increased *in vitro* antibacterial activity and minimal cytotoxicity (Bahar and Ren, 2013; Lee et al., 2019), and recently, peptides with lower cationicity have been associated with *in vivo* activity (Mishra et al., 2019).

Our approach of conducting data analysis on a pre-selected sub-set of AMPs with activity against Gram-negative bacteria to determine trends and frequency of amino acids resulted in novel AMPs with lab-tested *in vitro* activity against drug-resistant strains of bacteria. This combination of *in silico* design and in laboratory testing to validate the *in silico* predictions has proven highly successful. This approach can be used to design a variety of antimicrobial peptides against different strains or classes of bacteria, viruses and fungi to result in novel AMPs with a narrow spectrum of activity. Using a larger library of peptides may aid in designing variations of AMPs with predicted narrow and/or broad-spectrum of activity to further validate our design approach. Although our peptides demonstrated activity against MDR *E. coli*, they still need to be further tested against other strains of Gram-negative bacteria to determine the full range of their activity. PHNX AMPs effect on bacterial biofilms also needs to be assessed. Biofilms are a key virulence factor contributing to nosocomial infections and peptide therapeutics with activity against biofilm would be a breakthrough in modern medicine. The PHNX peptides also need some improvements in their hemolytic activity in future studies, perhaps through further rational design to improve their therapeutic index. Our study was a success as we designed AMPs with antibacterial activity against Gram-negative bacteria, supporting our overall design goal. Further testing *in vivo* with residue substitutions to improve antibacterial and hemolytic activity could result in improved AMPs. PHNX AMPs present novel synthetic peptide therapeutics which are a small step forward in developing new therapeutics to combat antibiotic resistance.

## Data Availability Statement

The original contributions presented in the study are included in the article/Supplementary Material, further inquiries can be directed to the corresponding author.

## Author Contributions

MV and SB conceived the design methodology. GW contributed database filtering technology and expertise. FA performed the laboratory experiments. All authors contributed to writing the manuscript and edited and approved the final version.

## Conflict of Interest

The authors declare that the research was conducted in the absence of any commercial or financial relationships that could be construed as a potential conflict of interest.

## Publisher’s Note

All claims expressed in this article are solely those of the authors and do not necessarily represent those of their affiliated organizations, or those of the publisher, the editors and the reviewers. Any product that may be evaluated in this article, or claim that may be made by its manufacturer, is not guaranteed or endorsed by the publisher.

## Footnotes

1
https://aps.unmc.edu/

2
https://heliquest.ipmc.cnrs.fr/cgi-bin/ComputParams.py

3
https://cbbio.online/AxPEP/

4
http://www.camp.bicnirrh.res.in/

5
http://www.bicnirrh.res.in/classamp/predict.php

6
https://dbaasp.org/prediction/general
