# Supplementary material for: Ab initio Designed Antimicrobial Peptides Against Gram-Negative Bacteria
Source: Front Microbiol. 2021 Nov 16;12:715246. doi: 10.3389/fmicb.2021.715246 (PMC8636942; doi:10.3389/fmicb.2021.715246)
Supplement: Supplementary file 1 [file Data_Sheet_1.docx]

# **Supplemental Information**

The secondary and tertiary structures of the AMPs were assessed using PEP2D (<http://crdd.osdd.net/raghava/pep2d/submit.html>) and PEP-FOLD3 (<https://bioserv.rpbs.univ-paris-diderot.fr/services/PEP-FOLD3/>) where the FASTA sequences was input, ran and the resulting predictions incorporated into **Table S1**(Singh, Singh, and Singh Raghava 2019; Lamiable et al. 2016). The amphipathicity was projected using a helical wheel via HeliQuest(Gautier et al. 2008). These predictions were conducted to determine the likelihood for the AMPs to penetrate the bacterial bilayer membrane for antimicrobial activity with the structures shown in **Table S1**. All PHNX AMPs were predicted to have a helix for at least part of the peptide. PHNX-4 and -5 predicted to form a helix close to the N-terminus. PHNX-8 predicted a secondary structure with helices, coils with a rana-box near the C-terminus and a tertiary structure with a N-terminal helix and a C-terminal coil. Given the diversity in the structures of naturally occurring AMPs, the synthetic PHNX AMPs are predicted to form helices mixed with coils with close similarity to the structures of exiting, naturally occurring AMPs; hence, it is likely the predicted structures will allow bacterial penetrance and membrane rupture *in*-*vitro*.

**Table S1.** Secondary and tertiary structures of the designed synthetic AMPs

| **Name** | **Tertiary Structure**(Lamiable et al. 2016) | **Secondary Structure**(Singh, Singh, and Singh Raghava 2019) | **Helical Wheel Projection** |
| --- | --- | --- | --- |
| PHNX-1 | 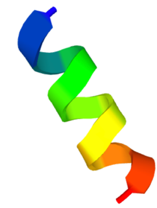 | 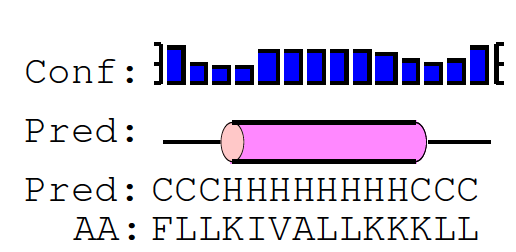 | 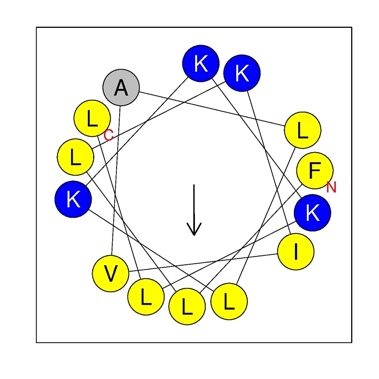 |
| PHNX-2 | 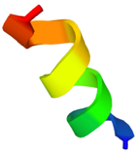 | 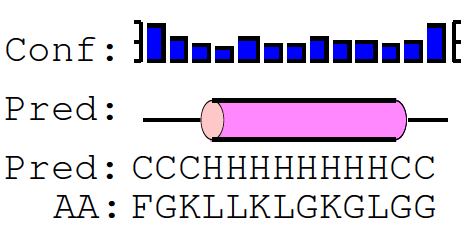 | 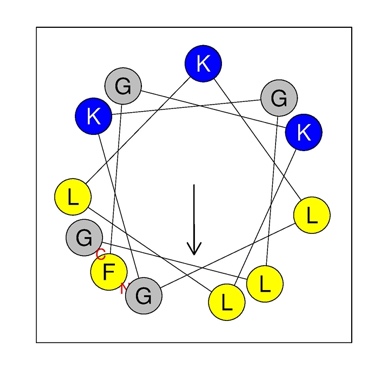 |
| PHNX-3 | 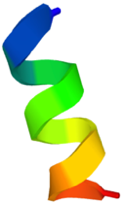 | 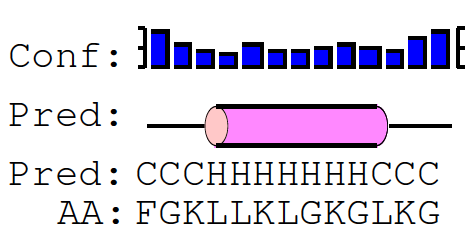 | 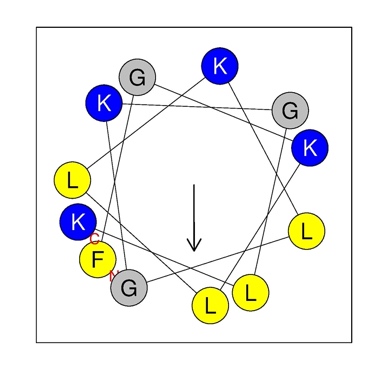 |
| PHNX-4 | 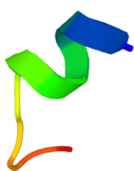 | 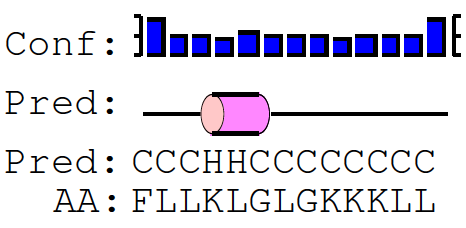 | 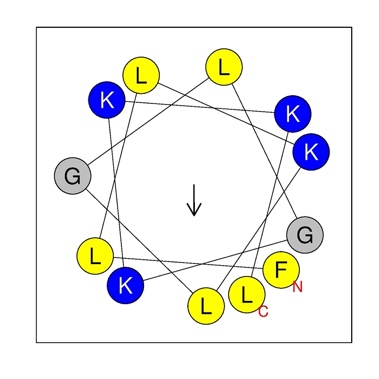 |
| PHNX-5 | 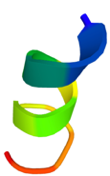 | 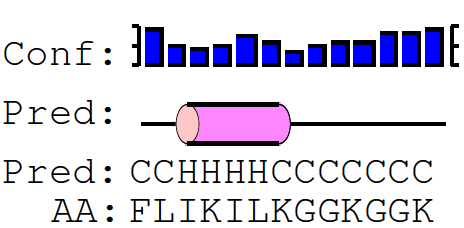 | 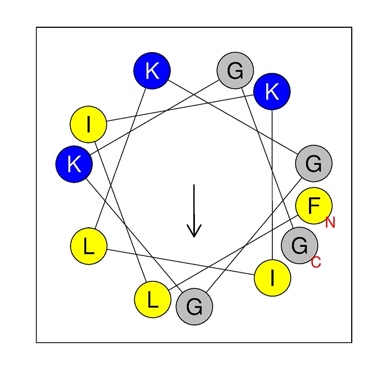 |
| PHNX-6 | 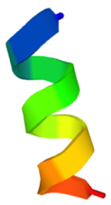 | 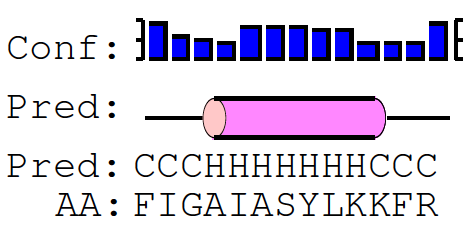 | 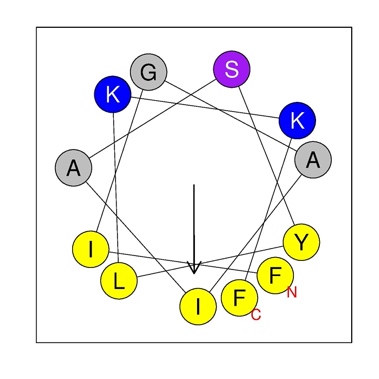 |
| PHNX-7 | 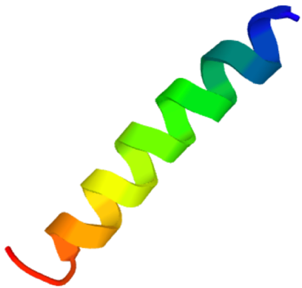 | 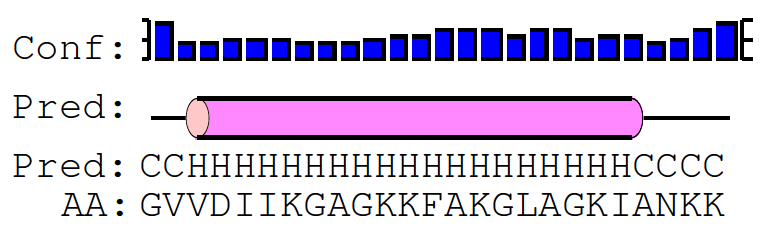 | 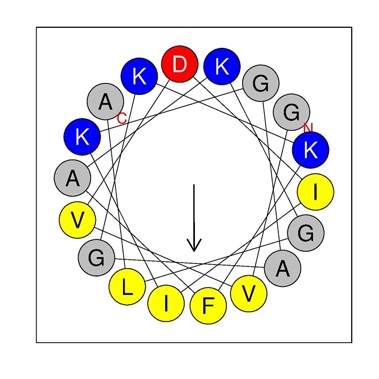 |
| PHNX-8 | 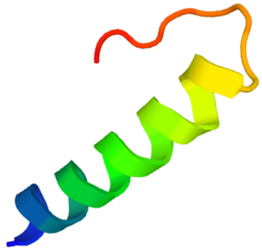 | 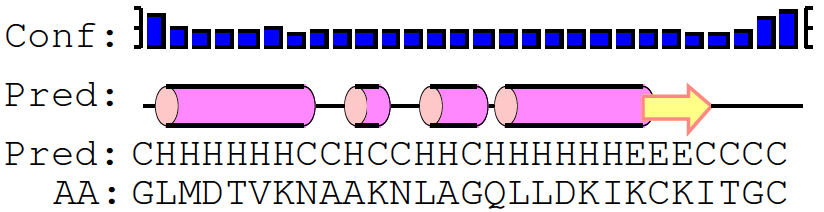 | 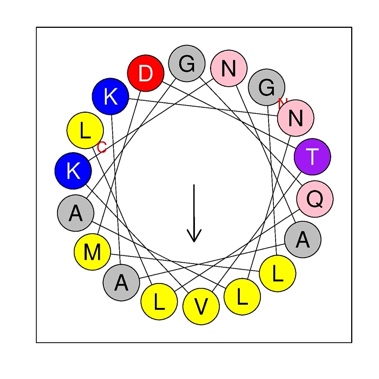 |

**Figure S1.** Statistics of AMP properties obtained from APD3. **A**. Lengths of AMPs in Dataset2. **B**. Frequency of total amino acid residues in Dataset2. **C**. Charge of the AMPs in Dataset2 **D**. Hydrophobicity percentage of AMPs in Dataset2 (Hydrophobicity was calculated as a percentage of hydrophobic amino acids, based on the Kyte and Doolite scale (Kyte and Doolittle 1982), divided by the total number of amino acids per AMP)
