# Supplementary figures and images for: Ab initio Designed Antimicrobial Peptides Against Gram-Negative Bacteria
Source: Front Microbiol. 2021 Nov 16;12:715246. doi: 10.3389/fmicb.2021.715246 (PMC8636942; doi:10.3389/fmicb.2021.715246)

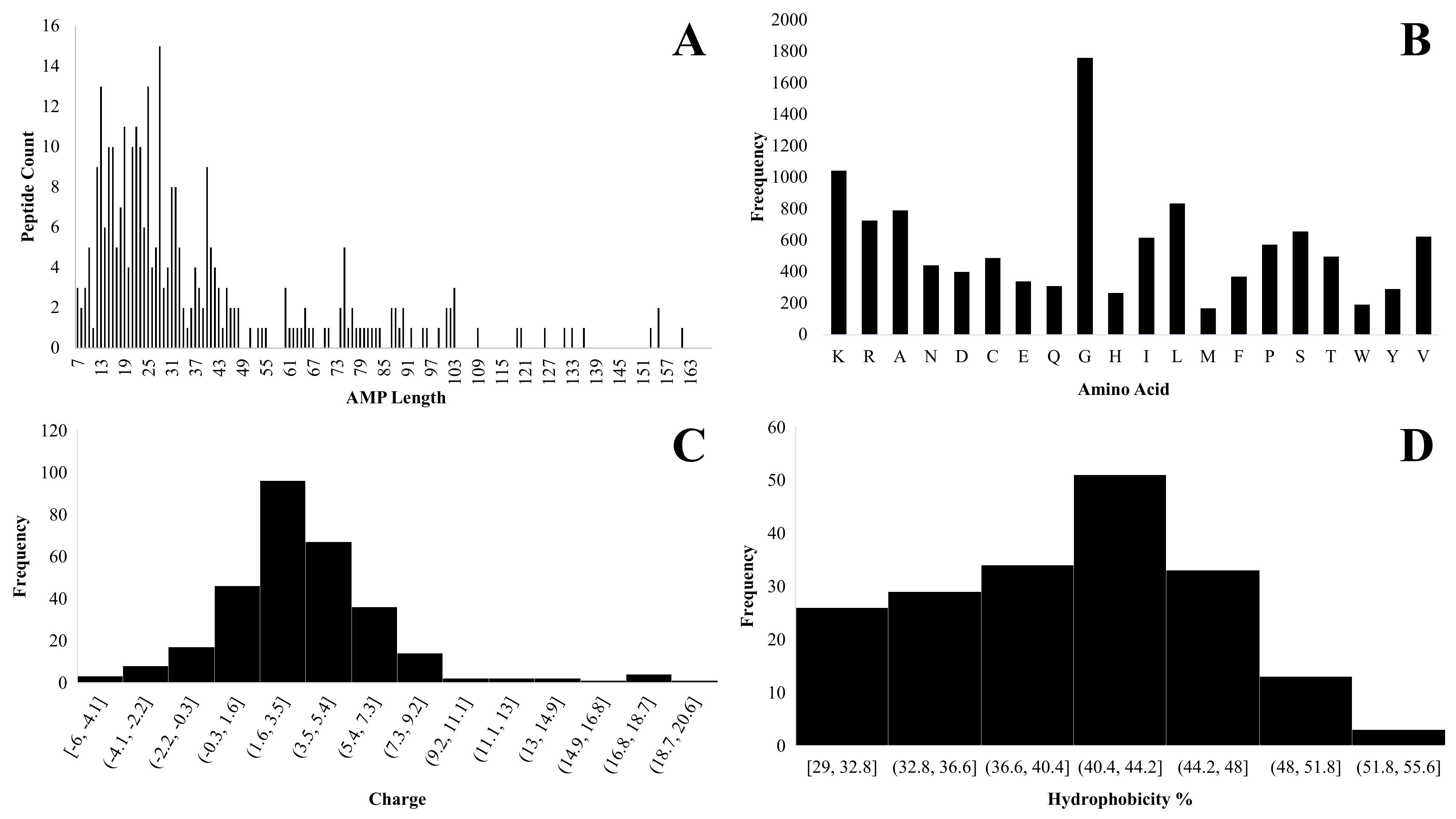

Supplement: Supplementary file 2 [file Image_1.TIFF]
